# The relationship between evaluation of shared decision-making by pet owners and veterinarians and satisfaction with veterinary consultations

**DOI:** 10.1186/s12917-022-03401-6

**Published:** 2022-08-02

**Authors:** Yuma Ito, Hirono Ishikawa, Asuka Suzuki, Mio Kato

**Affiliations:** Graduate School of Public Health, Teikyo University, 2-11-1, Kaga, Itabashi-ku, Tokyo, 173-8605 Japan

**Keywords:** Veterinary medicine, Veterinary communication, Shared decision-making, Decision-making preference

## Abstract

**Background:**

Communication skills are a necessary competency in veterinary medicine, and shared decision-making (SDM) between practitioners and patients is becoming increasingly important in veterinary practice as in human medicine. There are few studies that have quantitatively measured SDM in veterinary health care, and the relationship between SDM and consultation satisfaction is unknown. The purpose of this study was to investigate the status of SDM implementation in veterinary hospitals and the relationship between SDM implementation and consultation satisfaction among pet owners. We conducted a cross-sectional study using self-administered questionnaires among pet owners and veterinarians. In total, 77 pet owners who visited a veterinary clinic and 14 veterinarians at the clinics participated in this study. After a veterinary clinic visit, owners were asked to rate their decision-making preferences using the Shared Decision Making Questionnaire for patients (SDM-Q-9) adapted for veterinary medicine, as well as their satisfaction with the consultation. The corresponding veterinarians were asked to complete the veterinary version of the survey (SDM-Q-Doc).

**Results:**

Most pet owners (64.9%) preferred SDM in veterinary consultations. Cronbach's alpha coefficient of 0.84 for the veterinary SDM-Q-9 and 0.89 for the veterinary SDM-Q-Doc both confirmed high reliability. The Spearman's correlation coefficient between the SDM-Q-9 and consultation satisfaction was 0.526 (*p* < 0.001), which was significant. The SDM-Q-Doc was not significantly correlated with either the SDM-Q-9 or pet owner consultation satisfaction. We conducted a sensitivity analysis of correlations among veterinarians; responses on the SDM-Q-Doc to examine the association between the SDM-Q-Doc and SDM-Q-9 and owner satisfaction; the results remained the same and no association was found.

**Conclusions:**

Our findings suggest that evaluation of SDM among pet owners was associated with their satisfaction with veterinary consultation. Veterinarians may be able to improve the satisfaction level of pet owners by adopting a consultation method that increases SDM. We did not consider the content of veterinary care or the number of visits to the veterinary clinic; future studies should be conducted to confirm the validity of our results.

## Background

In 2021, there were 8,489,000 dogs and 9,644,000 cats in Japan, for a total of approximately 18,130,000 pets kept as companion animals [1]. In many households, animals such as cats and dogs are becoming irreplaceable members of the family, just like humans. The effects of pet ownership on human health have long been studied. A society that coexists with pets will be important for human public health in the future. For example, owning dogs and cats has been reported to reduce the risk of heart disease in humans as well as improve developmental delay in infants and mental health [2–5].

Veterinary care is a necessary part of pet ownership. As the importance of pets increases, so does the demand for good veterinary care. In veterinary medicine, communication skills are being recognized as an essential competency for veterinarians, in addition to clinical knowledge and skills. Previous studies have indicated that communication between veterinarians and pet owners is essential for better veterinary care [6–9].

In recent years, human medicine has emphasized respect for patients' values, facilitating patient participation in the treatment process and decision-making, and collaborating with patients. With growing acceptance of the concept of evidence-based medicine (EBM), shared decision-making (SDM) has been attracting attention as a way of building consensus between patients and health care providers.

EBM involves delivering optimal individual patient care by integrating the current best evidence on pathophysiological knowledge, cost-effectiveness, and patient preferences [10, 11]. SDM is the process of clinician and patient jointly participating in making a treatment decision after discussing the options, benefits, and harms, and considering the patient’s values, preferences, and circumstances [12]. SDM is considered to be the intersection of patient-centered communication skills and EBM to achieve optimal patient care [13]. It is believed that better decision-making can be achieved in situations of high uncertainty if health care providers are aware of SDM and work with patients to make decisions based on their knowledge of EBM [13]. SDM is also being studied in various departments, such as oncology and dermatology [14–16].The implementation of SDM has also been reported to have benefits such as increased satisfaction with consultations and reduced conflict in decision-making [17–19].

There are many similarities between communication in human and veterinary medicine, and the evidence on effective communication in human medicine is considered applicable to veterinary medicine [20]. The relationship between the veterinarian and the pet owner has often been based on paternalism; thus, the need for SDM has been proposed [21–23]. Although definitions of good medical care vary, it is commonly believed that appropriate SDM is important [24]. In particular, communication regarding end-of-life care, serious diseases, informing of negative situations, and euthanasia requires patient-centered communication skills for SDM [25–29]. Thus, communication skills training is necessary to achieve satisfactory communication. Communication training is needed in undergraduate veterinary education as well as in post-graduate education [30]. However, few quantitative studies have reported the current status of SDM implementation and its effectiveness in veterinary medicine. The scales developed to measure SDM in human medicine have been modified and validated for use in veterinary medicine [31, 32]. A previous study using the Observer OPTION5 instrument evaluated conversations between veterinarians and pet owners in clinic settings, and found that communication by veterinarians using SDM facilitated owner participation in decision-making [31].

Another study quantitatively assessed the implementation of SDM based on pet owners’ reflections after the death of their pet and suggested that the implementation of SDM was associated with a reduction in painful feelings of loss [33]. By analyzing the content of communication, veterinarians will be able to understand the preferences of pet owners and achieve good decision-making [32].

However, no studies have evaluated the implementation of SDM in specific consultations in veterinary medicine from the perspectives of both veterinarians and pet owners.

The purpose of this study was to investigate SDM implementation during veterinary consultations from the perspective of veterinarians and pet owners and to examine its relationship with owner satisfaction with the consultation. We examined the following research questions: (1) Is SDM expected by pet owners in veterinary medicine? (2) How do veterinarians and pet owners evaluate SDM in consultations? (3) Is the implementation of SDM in consultations related to owner satisfaction with the consultation?

## Methods

### Study population and setting

This was a cross-sectional study using self-administered questionnaires among pet owners and veterinarians. The questionnaires were distributed to owners who visited a veterinary clinic in Tokyo between July 5–7 and July 9–11 in 2021. This veterinary clinic is a relatively large private clinic in an area of the Tokyo metropolitan district whose residents have average-level incomes. The clinic receives referral cases from surrounding clinics. There are approximately 15 veterinarians working at the clinic, who only treat cats and dogs and consult in all specialties.

The first author, a veterinarian who had been working at this clinic, carried out participant recruitment and distribution of the questionnaires. All veterinarians working at the clinic were asked to participate in the survey and to sign a consent form. The questionnaires were distributed to owners who met either of the following two criteria: 1) completed a consultation where the veterinarian considered that a new decision had been made (as reported by the veterinarian), and 2) completed a consultation where a new medication was prescribed or a new treatment was provided (confirmed by the medical record). If the consultation met either of these criteria, the owner was recruited to participate in the study after the consultation was completed; owners were asked to sign a consent form and complete a questionnaire. A questionnaire was also distributed to the corresponding veterinarian who examined each pet whose owner participated in the survey. A veterinarian completed a questionnaire for each consultation included in the study. Consent forms and questionnaires were collected on the same day. Study participation and survey completion were on a voluntary basis. Participants were informed that they could withdraw from the study at any time, and owners were assured that their veterinarian would not be informed of their individual responses. No pilot study of this research has been conducted.

### Measures

The following variables pertaining to pet owners and veterinarians were obtained using the questionnaire.

### Decision-making preferences

Decision-making preferences were measured with an item used in previous studies on cancer care [34, 35]. We replaced the term “doctor” in the original item with “veterinarian”. Pet owners were asked if they prefer 1) the veterinarian to make the treatment decision on their own, 2) the veterinarian to make the treatment decision after talking them, 3) to make the treatment decision together with the veterinarian, 4) to make the treatment decision after hearing the veterinarian’s opinion, or 5) to make the treatment decision on their own.

According to the previous study, 1) and 2) were categorized as “passive,” 3) as “shared,” and 4) and 5) as “active” decision-making in the analyses [35].

### SDM

The implementation of SDM was measured using the Shared Decision Making Questionnaire for pet owners (SDM-Q-9) and the version for veterinarians (SDM-Q-Doc). Because some consultations may not involve any new decision-making (i.e., routine subcutaneous transfusion or only prescription of medications), we asked participants to complete the questionnaire only if there was new decision-making during the consultation.

Both the SDM-Q-9 and SDM-Q-Doc have a one-factor structure that consists of nine items; this structure measures the concept of SDM from the patient’s (SDM-Q-9) and the physician’s (SDM-Q-Doc) perspective. All nine items of both the SDM-Q-9 and SDM-Q-Doc are rated on a six-point Likert-type scale ranging, from 0 (Not applicable at all) to 5 (Very applicable). Total scores on each scale range from 0 to 45, with higher scores indicating a higher level of SDM. We followed the development procedure of the original version and transformed the sum scale to range from 0 to 100 points.

As of 2022, both questionnaires have been translated into 29 languages and their validity and reliability have been confirmed in various cultures and languages [36–40]. The Japanese version of the SDM-Q-9 has been shown to be reliable and valid [40]. The SDM-Q-9 and SDM-Q-Doc are also available in various versions for adaptation to other health care professionals and pediatric parents [41, 42]. The SDM-Q-9 and SDM-Q-Doc have been applied in veterinary medicine by Testoni et al. [33, 36]. Permission to change the terms “doctor” to “veterinarian” and “patient” to “pet owner” to fit the veterinary context in translation of the questionnaire was obtained from the authors of the original study (January 27, 2021).

### Satisfaction with consultation

We used a scale from a study that investigated the satisfaction level of outpatients in Japan [43], replacing the term “doctor” with “veterinarian.” The scale consists of four items addressing patient satisfaction with the consultation, which are rated on a five-point Likert-type scale ranging from 1 (Not applicable at all) to 5 (Very applicable). Total scores on each scale ranged from 4 to 20, with higher scores indicating greater satisfaction.

### Characteristics of survey respondents

The following variables were obtained as part of the questionnaire for pet owners: the owner's sex, age, education level, living situation, other animal ownership. We also queried their pet’s species, sex, age, health insurance status, and number of previous consultations.

The following variables were obtained as part of the questionnaire for veterinarians: sex, age, and years of clinical experience.

### Sample size

In human medical care, the correlation coefficients of previous studies that examined the correlation between the SDM-Q-9 and consultation satisfaction ranged from 0.28 to 0.39 [17].

Assuming a correlation coefficient of 0.35 in veterinary medicine, with α = 0.05 (two-sided) and power = 0.9 (β = 0.1), the sample size is 82; with α = 0.05 (two-sided) and power = 0.8 (β = 0.2), the sample size is 67. The sample size was calculated using SAS version 9.4 (SAS Institute Inc., Cary, NC, USA).

### Statistical analysis

Descriptive statistics were used for responses on the SDM-Q-9, SDM-Q-Doc and for pet owners' satisfaction as adapted veterinary medicine. Distributions were confirmed using the Shapiro–Wilk test.

Pearson's or Spearman's correlation coefficients were calculated for the association between SDM-Q-9 and SDM-Q-Doc, SDM-Q-9 and pet owners' satisfaction, and SDM-Q-Doc and pet owners' satisfaction. We used Spearman's correlation coefficient when scores were considered non-normally distributed according to the Shapiro–Wilk test.

The relationships among pet owners’ characteristics, decision-making preferences, responses on the SDM-Q-9 and SDM-Q-Doc, and pet owner’s satisfaction were analyzed using the Wilcoxon rank-sum test (for comparisons between two groups) or Kruskal–Wallis test (for comparisons between more than two groups). To identify factors strongly associated with satisfaction, variable selection was carried out using a stepwise method in a linear regression model, with pet owners’ satisfaction as the response variable and sex, age, education, life circumstances, owning other dogs or cats, number of consultations, and characteristics of pets as explanatory variables.

The following analyses were also performed as sensitivity analyses. The correlation between the veterinarian SDM scores and pet owner satisfaction (or owner SDM scores) was analyzed using a two-step approach considering clustering of SDM scores for veterinarians because the correlation coefficient does not account for similar responses from the same veterinarian. Slopes were estimated using a simple regression with each veterinarian SDM score as a response variable and pet owner satisfaction (or owner SDM score) as an explanatory variable, followed by the Z-test with a null hypothesis of the mean slope being zero.

The statistical significance for all tests was defined as *p* < 0.05. All analyses were performed using SAS version 9.4.

### Ethical considerations

The Teikyo University Ethical Review Board for Medical and Health Research Involving Human Subjects approved this study after ethical review (approval No. 21–001). All procedures performed in studies involving human participants were conducted according to the ethical standards of the institution and those of the 1964 Declaration of Helsinki and its later amendments or comparable ethical standards.

## Results

### Study participants

During the study period, 79 pet owners were recruited and 77 provided their consent for participation (response rate, 97.5%). The main reason for refusal to participate was the owner having difficulty reading the questionnaire because of deteriorating eyesight. The sample size did not reach the target with power = 0.9 because fewer consultations than expected met the inclusion criteria. Nevertheless, the sample size reached the target size calculated with power = 0.8.

**Fig. 1:**
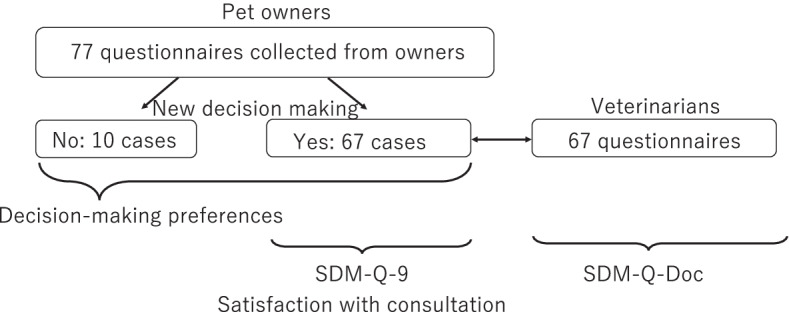
Flow of participants in the study. SDM-Q-9, Shared Decision Making Questionnaire – pet owner; SDM-Q-Doc, Shared Decision Making Questionnaire – veterinarian

Among the 77 pet owners who participated in this study, 67 stated that there was new decision-making during consultation and completed the SDM-Q-9. Therefore, responses for 67 SDM-Q-Doc questionnaires were also obtained from the corresponding veterinarians who conducted the consultation. The flow of the research participants is shown in Fig. 1.

Most pet owners (65.0%) were in their 40 s or 50 s. The proportion of women (74.0%) was higher than that of men (26.0%). The highest level of educational attainment was high school and above for 44.2% of the study population, and university for 54.6%. Among animals treated, 72.7% were dogs and 27.3% were cats, of which 39.0% had health insurance. The clinical and demographic sample characteristics of pet owners and pets are displayed in Table 1.

**Table 1:** Characteristics of study participants: pet owners and pets

	Overall
	*n* = 77
Characteristics of pet owners	
Sex	
Male	20 (26.0%)
Female	57 (74.0%)
Age (y)	
Teens	2 (2.6%)
20 s	3 (3.9%)
30 s	8 (10.4%)
40 s	20 (26.0%)
50 s	30 (39.0%)
60 s	10 (13.0%)
70 s and over	4(5.2%)
Education	
Junior high school graduate	1 (1.3%)
High school graduate	34 (44.2%)
Junior college graduate	26 (33.8%)
University graduate or above	16 (20.8%)
Life circumstances	
Easy	2 (2.6%)
Somewhat easy	14 (18.2%)
Normal	57 (74.0%)
Somewhat difficult	3 (3.9%)
Difficult	1 (1.3%)
Own other dogs or cats	
Yes	34 (44.2%)
No	43 (55.8%)
Characteristics of pets	
Animal	
Dog	56 (72.7%)
Cat	21 (27.3%)
Sex	
Male, intact	19 (24.7%)
Male, neutered	16 (20.8%)
Female, intact	13 (16.9%)
Female, neutered	29 (37.7%)
Age (y)	
1	13 (16.9%)
2–5	7 (9.1%)
6–9	10 (13.0%)
10–13	25 (32.5%)
14–17	19 (24.7%)
18‐	3 (3.9%)
Insured	
Yes	30 (39.0%)
No	47 (61.0%)
Number of consultations	
1	6 (7.8%)
2–5	17 (22.1%)
6–10	15 (19.5%)
11–19	6 (7.8%)
20–	33 (42.9%)

Of the 14 veterinarians who participated in the study, 7 (50.0%) were men. Five veterinarians (35.7%) were in their 20 s, 6 (42.9%) in their 30 s, 2 (14.3%) in their 40 s, and 1 (7.1%) in their 50 s. The years of clinical experience was 1–3 years in 6 (42.9%) veterinarians, 4–7 years in 2 (14.3%), 8–11 years in 3 (21.4%), 12–15 years in 1 (7.1%), and 21–30 years in 2 (14.3%). Per veterinarian, the mean number of consultations with pet owners who completed the SDM-Q-9 was 4.8, ranging from 1 to 9.

### Decision-making preferences

As shown in Table 2, most pet owners (64.9%) expressed a strong preference for SDM, with 10% preferring to be actively involved in decision making, and 25% preferring that the veterinarian determine the treatment.

**Table 2:** Preferences of pet owners (*n* = 77)

	Overall
	*n* = 77
I prefer to make the treatment decision on my own	0 (0%)
I prefer to make the treatment decision after hearing the veterinarian’s opinion	8 (10.4%)
I prefer to make the treatment decision together with the veterinarian	50 (64.9%)
I prefer that the veterinarian make the treatment decision after talking with me	19 (24.7%)
I prefer the veterinarian to make the decision on their own	0 (0%)

### Evaluation of consultations according to the SDM-Q-9 and SDM-Q-Doc for veterinary medicine

We analyzed the total number (67) of completed SDM-Q-9 and SDM-Q-Doc questionnaires. The minimum value, maximum value, mean, median, and standard deviation (SD) of each item are presented in Table 3. The Cronbach’s α coefficient of the veterinary version of the SDM-Q-9 was 0.84; this was 0.89 for the SDM-Q-Doc. Both pet owners and veterinarians reported the highest scores on item 9 and the lowest on item 7. In general, veterinarians' ratings tended to be lower than those of pet owners. No significant difference was found in total scores on the SDM-Q-9 and SDM-Q-Doc according to pet owners’ demographic characteristics, pets’ characteristics, and owners’ decision-making preferences in the Wilcoxon rank-sum test or Kruskal–Wallis test. The correlation between the veterinary version of the SDM-Q-9 and the veterinary version of the SDM-Q-Doc was not statistically significant, with *r* = 0.095 (*p* = 0.447).

**Table 3:** Item characteristics of the SDM-Q-9 and SDM-Q-Doc adapted for veterinary medicine (*n* = 67)

		Pet owners				Veterinarians			
		Min	Max	Mean (median)	SD	Min	Max	Mean (median)	SD
SDM1	・My veterinarian made clear that a decision needs to be made ・I made clear to the pet owner that a decision needs to be made	2.22	11.11	9.95 (11.11)	1.66	0	11.11	8.92 (8.89)	2.46
SDM2	・My veterinarian wanted to know exactly how I want to be involved in making the decision ・I wanted to know exactly from the pet owner how they want to be involved in making the decision	4.44	11.11	9.79 (11.11)	1.51	2.22	11.11	8.29 (8.89)	2.52
SDM3	・My veterinarian told me that there are different options for treating my pet's medical condition ・I told the pet owner that there are different options for treating their pet's medical condition	0	11.11	9.33 (11.11)	2.29	2.22	11.11	7.69 (8.89)	2.74
SDM4	・My veterinarian precisely explained the advantages and disadvantages of the treatment options ・ I precisely explained the advantages and disadvantages of the treatment options to the pet owner	0	11.11	9.10 (8.89)	2.68	0	11.11	7.06 (6.67)	2.79
SDM5	・My veterinarian helped me understand all the information ・I helped the pet owner understand all the information	6.67	11.11	9.82 (11.11)	1.56	6.67	11.11	8.86 (8.89)	1.71
SDM6	・My veterinarian asked me which treatment option I prefer ・I asked the pet owner which treatment option they prefer	2.22	11.11	9.36 (11.11)	2.36	2.22	11.11	8.09 (8.89)	2.78
SDM7	・My veterinarian and I thoroughly weighed the different treatment options ・The pet owner and I thoroughly weighed the different treatment options	0	11.11	8.47 (8.89)	2.45	0	11.11	5.84 (6.67)	2.92
SDM8	・My veterinarian and I selected a treatment option together ・The pet owner and I selected a treatment option together	0	11.11	9.16 (8.89)	2.03	0	11.11	7.83 (8.89)	2.77
SDM9	・My veterinarian and I reached an agreement on how to proceed ・The pet owner and I reached an agreement on how to proceed	4.44	11.11	10.15 (11.11)	1.5	4.44	11.11	9.45 (8.89)	1.66

*SDM-Q-9* Shared Decision Making Questionnaire – pet owner, *SDM-Q-Doc* Shared Decision Making Questionnaire – veterinarian, *SD* Standard deviation

### Relationship between SDM evaluation and satisfaction with veterinary consultation

The mean score for pet owners' satisfaction with the consultation was 18.82; the median was 20, 25th percentile was 18, and the 75th percentile was 20. Spearman's correlation coefficient between pet owners’ satisfaction with the consultation and SDM-Q-9 total score was 0.526 (*p* < 0.001), indicating a significant positive correlation. No statistically significant correlation was found between pet owners’ satisfaction with the consultation and the SDM-Q-Doc total score (*r* =  − 0.020, *p* = 0.866).

A multiple regression analysis using the stepwise method was conducted with pet owners’ satisfaction as the outcome variable and pet owners’ attributes, SDM-Q-9 score, and decision-making preference as explanatory variables. The only variable selected in the model was the SDM-Q-9 score.

Although we examined correlations among veterinarians’ responses, the results remained the same. The results of a Z-test to determine whether the mean of the slope estimates for each veterinarian was greater than zero showed that the mean (standard deviation, SD) of the slope estimates was 1.8 (3.2), with a *p*-value of 0.29 (or 0.45 [0.36], *p* = 0.11); no statistically significant positive correlation was found.

## Discussion

This was the first study to quantitatively investigate the implementation of SDM using the SDM-Q-9 and SDM-Q-Doc in veterinary medicine in Japan.

First, most pet owners preferred SDM in decision-making during veterinary consultations, which is similar to a previous study [23]. Therefore, implementation of SDM is as important and necessary in veterinary medicine as in human medical care. However, compared with studies conducted in human medicine, a higher proportion of pet owners preferred passive rather than active decision-making [34]. This might be partly because, in veterinary medicine, owners do not have much medical knowledge regarding animals; therefore, an attitude of "leaving it up to the veterinarian," who has more specialized knowledge about animals, may be more common in veterinary than in human medicine. Additionally, because we did not investigate the specific contents of veterinary examinations, it is possible that we included examinations in which the need for SDM was low. It will be necessary to examine in what situations SDM is necessary for veterinary medicine.

Second, this study revealed how veterinarians and owners evaluate SDM in veterinary consultations. The SDM-Q-9 and SDM-Q-Doc used in our study were both modified for use in veterinary medicine. Both instruments have high alpha coefficients, indicating that they are as reliable as the original scales [36, 39, 44]. As with other SDM scales, this scale was found to be applicable in veterinary medicine [24].Both the SDM-Q-9 and the SDM-Q-Doc show trends in the scores for each item similar to those in studies conducted among outpatients in Japan, with item 9 being the highest and item 7 the lowest [40]. This suggests that whereas SDM performed during consultation in veterinary medicine is similar to SDM in human medical care, SDM items that are difficult to achieve may also be similar. Additionally, there was no significant correlation between veterinarian and pet owner ratings of SDM in the same consultation. This is consistent with previous studies in human medical care showing no significant correlation between physicians' ratings of SDM and patients' ratings of SDM [39]. This suggests that in both human medicine and veterinary medicine, medical professionals who provide care and clients (pet owners) who receive the care provided might perceive SDM differently. Thus, it is important to evaluate SDM from both perspectives.

Third, in analysis of the relationship between SDM evaluation and satisfaction with the consultation, we found a significant relationship between SDM-Q-9 and consultation satisfaction, similar to previous studies in human medicine [17]. In veterinary medicine, the implementation of SDM may also be related to pet owners' satisfaction with their consultation. However, there was no significant correlation with SDM as evaluated by veterinarians and pet owner satisfaction with the consultation. The fact that pet owners themselves reported that SDM was implemented in the consultation may be directly linked to a high level of satisfaction. As mentioned earlier, veterinarians and owners may have different criteria for evaluating SDM, and veterinarians need to understand how owners evaluate SDM in a consultation.

The results of this study will help future decision-making in the form of SDM to ensure that pet owners are satisfied with the decisions made regarding their pet. Communication skills training for veterinarians that includes SDM content, as is done in human medicine, is considered to be important [44]. It is necessary to develop the curriculum of veterinary schools to include communication skills training for SDM.

The limitations of this study are that it is a single-center study and the sample size was not large. The fact that the sample size did not reach the target during the research period may be owing to the small number of consultations that fit the inclusion criteria of this study. Communication methods and veterinary consultations may differ from country to country; therefore, our findings may not be generalizable to other countries. Because this was a cross-sectional study, it is not possible to determine causal relationships. It may be possible to increase the internal validity of the study by conducting investigations at multiple facilities or by increasing the sample size. A preexisting relationship of some owners with their veterinarian may have affected evaluation of the consultation. Because no inclusion criteria were set for the content of consultation, the evaluation may have been affected by the content and severity of the pet’s disease. Further study with first-time consultations or specific diseases such as oncology may be needed to explore the external validity of the results. Finally, pet owners only completed one survey, but veterinarians completed multiple questionnaires, which may have caused evaluation bias among veterinarians [14–16, 39].

## Conclusions

In this study, we investigated the implementation of SDM in veterinary consultations from the perspectives of both pet owners and veterinarians and examined its relationship with consultation satisfaction. We confirmed that most pet owners preferred SDM in decision-making during veterinary consultation, and the process of SDM was implemented in veterinary medicine similar to the process reported in previous studies of human medical care.

Our findings also suggested that implementation of SDM in veterinary consultations may lead to greater satisfaction among pet owners because the evaluation of SDM by owners was related to their satisfaction with consultations. Based on these findings, it is necessary to develop educational programs for veterinarians to help them understand SDM and acquire the skills to implement SDM in practice.

## Data Availability

The datasets used and/or analyzed during the current study are available from the corresponding author on reasonable request.

## Abbreviations

SDM: Shared decision making
EBM: Evidence-based medicine
